# Combined assessment of early and late‐phase outcomes in orphan drug development

**DOI:** 10.1002/sim.8952

**Published:** 2021-04-04

**Authors:** Konstantinos Pateras, Stavros Nikolakopoulos, Kit C. B. Roes

**Affiliations:** ^1^ Department of Data Science and Biostatistics Julius Center for Health Sciences and Primary Care, University Medical Center Utrecht Utrecht The Netherlands; ^2^ Department of Health Evidence Section Biostatistics, Radboud University Medical Centre Nijmegen The Netherlands

**Keywords:** Bayesian, bias correction, biomarker, borrowing strength, decision‐induced bias, rare diseases, surrogate endpoint, trial combination

## Abstract

In drug development programs, proof‐of‐concept Phase II clinical trials typically have a biomarker as a primary outcome, or an outcome that can be observed with relatively short follow‐up. Subsequently, the Phase III clinical trials aim to demonstrate the treatment effect based on a clinical outcome that often needs a longer follow‐up to be assessed. Early‐phase outcomes or biomarkers are typically associated with late‐phase outcomes and they are often included in Phase III trials. The decision to proceed to Phase III development is based on analysis of the early‐Phase II outcome data. In rare diseases, it is likely that only one Phase II trial and one Phase III trial are available. In such cases and before drug marketing authorization requests, positive results of the early‐phase outcome of Phase II trials are then likely seen as supporting (or even replicating) positive Phase III results on the late‐phase outcome, without a formal retrospective combined assessment and without accounting for between‐study differences. We used double‐regression modeling applied to the Phase II and Phase III results to numerically mimic this informal retrospective assessment. We provide an analytical solution for the bias and mean square error of the overall effect that leads to a corrected double‐regression. We further propose a flexible Bayesian double‐regression approach that minimizes the bias by accounting for between‐study differences via discounting the Phase II early‐phase outcome when they are not in line with the Phase III biomarker outcome results. We illustrate all methods with an orphan drug example for Fabry disease.

## 1 INTRODUCTION

Drug development programs typically include exploratory (Phase II) and confirmatory (Phase III) randomized controlled trials (RCTs) to assess the efficacy, safety and appropriate dosages of an experimental (new) treatment. For regular “large disease” drug development programs decisions to conduct a Phase III trial are based on positive Phase II trials. If these trials are only retrospectively evaluated in combination, that is, during the drug marketing authorization request, the ad hoc synthesis may induce a form of decision‐induced bias (the succeeding trials are only conducted when the first trials were positive). Such a bias is not an issue if the early and late Phase trials are prospectively considered in the design phase (eg, a seamless approach).

However, it is not uncommon that in rare diseases, no more than two independent RCTs are conducted and available, one exploratory and one confirmatory.^1^ Phase II primary endpoints are typically biomarkers or surrogate outcomes.^2^ Phase III primary clinical outcomes are likely established endpoints and they may either require (1) larger sample sizes, (2) more costly collection, (3) to be observed after a considerable time, or (4) be more variable outcomes than early‐phase outcomes, therefore, even if *N* = *N*_2_ + *N*_3_ number of patients participate in both trials, only *N*_3_ patients will be available to provide responses for the primary clinical outcome of interest. Biomarkers (early‐phase) and secondary clinical outcomes are often observed earlier and, therefore, easily included in both trials and, hence, available for all *N* patients. After both trials have been conducted, inference on the treatment efficacy is typically performed by evaluating the late‐phase outcome responses of *N*_3_ patients. In a rare disease setting, *N*_3_ may not be large enough to solidly confirm treatment efficacy. In assessing the totality of evidence, the positive results from the Phase II trial could *retrospectively* be seen as supportive, even if the two clinical trials were designed/conducted independently, as typically the early‐phase outcome would be assumed to be associated with the late phase primary clinical outcome. Throughout the article the terms “*retrospective (ly)*” denote the retrospective combination of the available Phase II and Phase III trial after both trials are completed and their final results are available.

For example, Galafold (migalastat) acquired marketing authorization as an orphan drug for the treatment of Fabry disease in 2016 within Europe. Fabry disease is a rare, progressive disorder with an estimated prevalence of 1:117 000 to 1:40 000.^3^ The condition affects major organs and may result in life‐threatening events. Until then, standard treatment for Fabry disease consisted of Enzyme Replacement Therapy.^3^ Two main studies were submitted during the marketing authorization of migalastat; one randomized, placebo‐controlled (AT1001‐011, migalastat vs Placebo) superiority study and one active comparison randomized trial (AT1001‐012, migalastat vs Enzyme Replacement Therapy), with a noninferiority design.

In trial 011 patients switched to migalastat 6 months postrandomization, while in trial 012 primary follow‐up was considerably longer, with switching taking place 18 months postrandomization. In the first trial, the change in average globotriaosylceramide (GL‐3) inclusions from baseline to 6 months was the primary outcome which produced nonconclusive evidence. The second trial utilized the annualized change in glomerular filtration rate (*eGFR*) at month 18 as primary clinical outcome (Table 1). Both GL‐3 and the annualized change in *eGFR* at month 6 were collected in both trials (011 and 012). No strong correlation has been established in the literature between the GL‐3 outcome and the change in glomerular filtration rate (*eGFR*).^4^

**TABLE 1:** Main randomized studies described in the European Public Assessment Report of Galafold

Study number	Duration	Annualized rates of change in *eGFR* from baseline to month 6	Annualized rates of change in *eGFR* from baseline to month 18	Sample size	Start date
AT1001‐011	6 months	Collected	Not collected	67	August 2009
AT1001‐012	18 months	Collected	Collected	52	December 2010

In study 011 after 6 months of treatment with migalastat 150 mg, eGFR values increased, whereas in the placebo treated group eGFR values declined.^3^ This outcome among other secondary results led to the conduct of study 012. In trial 011, all patients treatment switched to migalastat at 6 months, an action that restricts the observation of a treatment effect on the primary late‐phase outcome. Given the limited available data, evidence from both trials were retrospectively (ad hoc) assessed for the final approval decision.

Analysis methods that use the relation between early and late‐phase outcomes may be applied to retrospectively, but formally, synthesize the evidence on treatment efficacy across the two trials. Engel and Walstra^5^ formulated a *double‐regression* (DR) approach, which can aid in more precise treatment effect estimation, by accounting for unobserved late‐phase outcome responses via observed early‐phase outcome responses. Their method utilizes the correlation to ultimately inform the mean and variance estimates of the treatment effect on the late‐phase outcomes. For large samples their method has the potential to increase precision. However, for small sample sizes this is not necessarily true.^6^ Previously, in RCTs the DR approaches have been suggested mainly to inform treatment selection during interim analysis in seamless Phase II/III designs.^7^, ^8^, ^9^ Double‐regression methods can be even generally applied wherever there is possibility to include early outcome information in decision making during the course of a trial.^10^

A Bayesian *double‐regression* (BDR) analogue can be readily constructed^11^ which maintains similar limitations to the frequentist alternative but could flexibly model the two Phase III outcomes' data. Such a model can include historical trial data (ie, Phase II early‐phase outcome data or external information on the early and late‐phase outcome correlation) as a elicited prior distributions.^12^ Furthermore, this Bayesian model accounts for the uncertainty around each parameter during the borrowing of information.

In this article, we investigate how to model and estimate the efficacy of a new treatment on the late‐phase clinical outcome, using data on early‐phase outcomes from both trials. Most literature on double‐regression focuses on design aspects such as interim analysis or seamless design of phase II/III trials, though, in the present article we propose methods that would be applied retrospectively (ad hoc) only after the Phase III trial. We propose and investigate methods that either account or do not account for the potential decision‐induced bias when combining retrospectively the Phase II and Phase III trials. We investigate the two proposed models, the bias corrected DR approach and the flexible Bayesian approach regarding their performance to estimate the treatment effect on the late‐phase outcome. We focus on two related key problems: (1) the magnitude of the type 1 error inflation when retrospectively combining data from Phase II and III and (2) how to estimate the treatment effect on the late‐phase outcome, using results from both studies and we assess this estimate in terms of bias and variance.

The article is organized as follows. First, we describe a bivariate linear model, we introduce its conditional form and we formalize the (often visual) retrospective pooling by utilizing DR with nonavailable Phase II late‐phase outcome data, then briefly discuss specific model variations, for example, the *single‐regression* (SR) approach. We introduce the problem of decision‐induced bias moving from Phase II to Phase III based on the Phase II early‐phase outcome in Section 3 and then provide an approximate analytical solution. In Section 4, we propose and formulate a Bayesian two‐step solution to the estimation problem, a model that down‐weights the impact of the biomarker data via a historical power prior. This prior dynamically accounts for the bias in estimating the same treatment effect across the two trials, by accounting for additional between‐trial differences (variability) around the biomarker outcome effect. The article ends with a discussion and steps for further research.

## 2 MODELS FOR THE JOINT PHASE II AND III DATA

Consider a Phase II trial of total sample size *N*_2_ and a Phase III trial of total sample size *N*_3_. For both trials it is assumed that a number of patients (*N*_*k*_ = *n*_*ck*_ + *n*_*ek*_,  *n*_*k*_ = *N*_*k*_/2,  *k* = 2, 3) are randomized to the control and experimental treatment. Let us denote *Y*_*ik*_ the late‐phase treatment response for patient *i* in trial *k* and *X*_*ik*_ the early‐phase treatment response for patient *i* in trial *k*, *k* = 2, 3,  *i* = 1, 2, … *N*_*k*_.

### 2.1 Bivariate modeling for early‐phase and late‐phase outcomes between studies

When all late‐phase *Y*_*i*_ = (*Y*_*i*2_, *Y*_*i*3_) and early‐phase *X*_*i*_ = (*X*_*i*2_, *X*_*i*3_) outcomes are available where *i* = 1, 2, … , *N*, they are assumed to follow a bivariate normal distribution as

(m1) $$\begin{array}{ll} \left(\begin{array}{c} {X_{i}}_{}\\ {Y_{i}}_{} \end{array}\right) & ∼\text{BVN}\left[\begin{array}{cc} \left(\begin{array}{c} a_{x}+b_{x}t_{i}\\ a_{y}+b_{y}t_{i} \end{array}\right), & \sum =\left(\begin{array}{cc} \sigma_{x}^{2} & \rho_{}\sigma_{x}\sigma_{y}\\ \rho_{}\sigma_{x}\sigma_{y} & \sigma_{y}^{2} \end{array}\right) \end{array}\right], \end{array}$$

where $\sigma_{x}^{2}$ and $\sigma_{y}^{2}$ denote the true outcomes variances, $\rho_{}$ the true correlation between the two outcomes and **t**_**i**_ a vector indicating whether the *ith* patient receives control or experimental treatment. For the remainder of the article we drop index *i* to aid readability.

The above bivariate model can be conditionally expressed as

(m2) $$\begin{array}{ll} X_{}|t & ∼N(a_{x}+b_{x}t,\sigma_{x}^{2})\\ Y_{}|t,x & ∼N(a_{0}+b_{0}t+\gamma x,\sigma_{0}^{2}), \end{array}$$

where $\sigma_{0}^{2}=\sigma_{y}^{2}-\gamma^{2}\sigma_{x}^{2}$, $a_{0}=a_{y}-\gamma a_{x}$, $b_{o}=b_{y}-\gamma b_{x}$, and $\gamma =\rho_{}\sigma_{y}/\sigma_{x}$

### 2.2 Double regression to estimate the effect of primary late‐phase outcome

**FIGURE 1:**
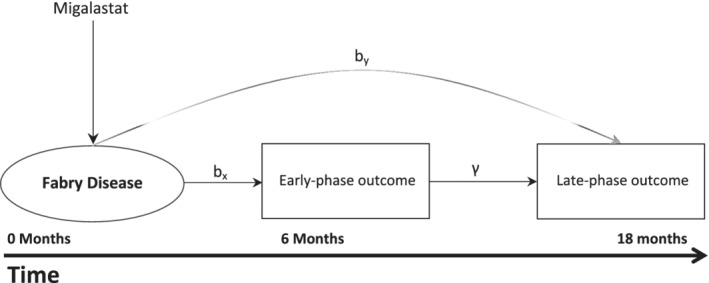
Relation between treatment vs early‐phase outcome, treatment vs late‐phase outcome and early‐phase vs late‐phase outcome in the example of Fabry disease

At the end of both trials early‐phase outcome data *X* for *N* = *N*_2_ + *N*_3_ patients and late‐phase outcome data *Y*_3_ for only *N*_3_ patients are observed. As *Y*_2_ is not observed, *Y* = *Y*_3_ and *X* = (*X*_2_, *X*_3_) now denote the observed late‐phase and early‐phase outcome data which correspond to patients of Phase II and Phase III trials. *Y* corresponds to the outcome of interest related to which estimation and hypothesis testing will be performed in *N*_3_ patients. The DR utilizes the relation between early‐phase and late‐phase outcomes and allows estimation of the main parameter of interest, the treatment effect on the late‐phase clinical outcome, *b*_*y*_ (Figure 1).

Based on the DR method, parameters *a*_*x*_, *b*_*x*_, and $\sigma_{x}^{2}$ are estimated via the regression of *X*|**t** on *N* patients, as ${â}_{x},{\hat{b}}_{x}$, $s_{x}^{2}$ and parameters $a_{0},b_{0},\gamma$, and $\sigma_{0}^{2}$ are estimated via the regression of *Y*_3_|*X*_3_, **t** on *N*_3_ patients, as ${â}_{0},{\hat{b}}_{0},\hat{\gamma }$, $s_{0}^{2}$, while $s_{y}^{2}=s_{0}^{2}+{\hat{\gamma }}^{2}s_{x}^{2}$, ${â}_{y}={â}_{0}+\hat{\gamma }{â}_{x}$, $\hat{\rho }=\hat{\gamma }s_{x}/s_{y}$.^5^, ^8^ The primary effect of interest *b*_*y*_ is then estimated as:

(eq1) $$\begin{array}{ll} {\hat{b}}_{y}={\hat{b}}_{o}+\hat{\gamma }{\hat{b}}_{x}. & \end{array}$$

The variance of ${\hat{b}}_{y}$ is shown in Reference 5 to be equal to

$$\mathrm{var}({\hat{b}}_{0})+{\hat{\gamma }}^{2}\;\mathrm{var}({\hat{b}}_{x})+{\hat{b}}_{x}\;\mathrm{var}(\hat{\gamma })+2{\hat{b}}_{x}\;\text{cov}({\hat{b}}_{0},\hat{\gamma })$$

estimates of the above can be obtained by using the individual estimates acquired from the regression analyses (m2). Under model (m2), hypothesis testing is performed as *H*_0_ : *b*_*y*_ ≤ 0 vs *H*_1_ : *b*_*y*_ > 0 via $z_{1-\alpha_{3}}>{\hat{b}}_{y}/\sqrt{\hat{\mathrm{var}}(b_{y})}$, where $z_{1-\alpha_{3}}$ is the $(1-\alpha_{3})th$ standard normal quantile and $\alpha_{3}$ is the alpha level of the late‐phase primary outcome of phase III trial. A direct Bayesian analogue to the conditional model (m2) has been discussed elsewhere.^11^ Under diffuse “non‐informative” priors, this Bayesian model has been shown to produce comparable posterior means for all parameters to the estimates produced by model (m2).

### 2.3 Bayesian (double‐) regression

We can model the Phase II biomarker data (*X*_2_) via a Bayesian SR, $X_{2}|t∼N(a_{x}+b_{x}t,\sigma_{x}^{2}),a_{x},b_{x}∼N(0,10^{2}),\sigma_{x}^{2}∼\text{IG}(1,1)$ of *N*_2_ patients and we can utilize the posterior distribution Markov Chain Monte Carlo sample draws to construct a prior on a BDR model on the Phase III early‐phase outcome data as follows.

Let us assume a bivariate normal model for the biomarker and the primary late‐phase clinical outcome data *X*_3_ and *Y*_3_ corresponding to *N*_3_ patients with a covariance matrix ∑ as in model (m1). In our two‐dimensional scenario, a bivariate normal likelihood could be specified on the early‐phase and late‐phase Phase III outcome data by conditional distributions as follows

(m3) $$\begin{array}{ll} X_{3}|t & ∼N(a_{x}+b_{x}t,\sigma_{x}^{2})\\ a_{x} & ∼N(\mu_{ah},\sigma_{ah}^{2}),b_{x}∼N(\mu_{bh},\sigma_{bh}^{2})\\ \sigma_{x}^{2} & ∼\text{IG}(\alpha_{h},\beta_{h})\\ Y_{3}|t,x_{3} & ∼N(a_{y}+b_{y}t+\rho \frac{\sigma_{y}}{\sigma_{x}}x_{3},(1-\rho^{2})\sigma_{y}^{2})\\ a_{y} & ∼N(0,10^{2}),b_{y}∼N(0,10^{2})\\ \sigma_{y}^{2} & ∼\text{IG}(1,1)\\ \rho & ∼U(-1,1). \end{array}$$

The prior on ρ uniformly weights our prior considerations around the correlation parameter. In order to mimic model (m2) we have set normal distribution priors based on Phase II posterior effect and variance mean estimates of the early‐phase outcome parameters ($\mu_{ah},\mu_{bh},\sigma_{ah}^{2},\sigma_{bh}^{2}$). To further mimic model (m2) we inform the $\sigma_{x}$ prior based on the posterior model variance samples from Phase II early‐phase outcome data, that is, fitting them over an optimized gamma prior distribution, $\sigma_{x}^{2}∼G(\alpha_{h},\beta_{h})$. The above two‐step procedure will allow for possible discounting of the Phase II trial by down‐weighing the early‐phase historical outcome data, which is further discussed in section 4.

In comparison to the direct Bayesian analogue of model (m2), where the strength of the relationship between early and late‐phase endpoints becomes clear only after combining the posterior mean estimates via the γ parameter, model (m3) is more intuitive, as it directly models the correlation (ρ) between the two outcomes, and it directly produces posterior Markov Chain Monte Carlo draws from *b*_*y*_. Therefore, under such a fully Bayesian approach there is no need for numerical addition of treatment effect mean estimates.^11^ Posterior inference can be obtained via traditional Markov Chain Monte Carlo application software (ie, JAGS^13^) or even analytically under convenient prior distributions.^12^ In this Bayesian model we assume that hypothesis testing for *H*_0_ vs *H*_1_ will be performed by utilizing posterior probabilities as $Pr(b_{y}>0|Y_{})>\omega$ where ω=0.95.

If we set the correlation very close to zero; that is, ρ∼U(−0.01,0.01), then, the Phase III trial late‐phase outcome data are evaluated individually under a standard (Bayesian) linear SR model. In comparison to the SR models, the advantage of models (m2), Bayesian (m2) and (m3) rest in their ability to numerically calculate/imitate the impact of accounting for the Phase II early‐phase outcome data in analyzing the late‐phase outcome. Additional details of the *(Bayesian) SR* models can be found in Appendix A.

## 3 TYPE 1 ERROR INFLATION AND BIAS DUE TO SELECTION BASED ON EARLY‐PHASE OUTCOME RESULTS

**FIGURE 2:**
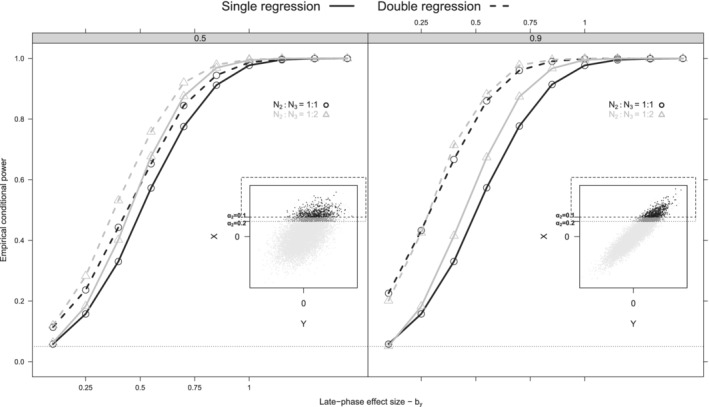
Conditional power curves comparing the performance of the single and double‐regression for the following scenarios; *N*_2_:*N*_3_ ∈ {1:1, 1:2}, $\sigma_{y}^{2}=\sigma_{x}^{2}=1$, $\rho_{r}\in \{0.1,0.9\}$, *N* = 120, $\alpha_{2}=0.1$, and *b*_*y*_, *b*_*x*_ ∈ {0, 0.1, 0.2, … , 1}. No between‐trial outcome variation was introduced in this set up and each scenario was replicated 10 000 times. The inner figures serve as an explanation to the observed type I error increase, as they present the joint strict null hypothesis (*b*_*y*_ = *b*_*x*_ = 0) distribution of the early and late‐phase treatment effect for the Phase III trials (light gray dots) and the truncated, based on a positive decision criteria, Phase II trials (black and dark gray dots). When utilizing the Phase II trials (darker dots in the inner Figures), larger critical levels result in an average overestimation of the treatment effect which consistently produces an average increase in error rates and on average larger bias is incorporated in the final inference. This mean increase can be observed in the expression of mean square error for the late‐phase treatment effect estimate (eq3). As expected based on (eq3), all error rates increase with higher ρ and the power curve increases with lower σ. A similar behavior was observed between the equivalent Bayesian single‐regression and Bayesian double‐regression alternative

The potential issues with retrospective combination of early and late phase results stem mostly from the fact that they are not independent. Usually, a Phase II decision leads to the initiation of a Phase III trial. This decision could be based on a test statistic for the early‐phase outcome and an imposed critical value; that is, $z_{1-\alpha_{2}}$. This is clearly an oversimplification of the actual Phase II to Phase III transition decision, but used here to illustrate the potential impact on type 1 error and bias if the results are retrospectively combined. In this simplified model, the distribution of the available Phase II trial early‐phase outcome $f(X_{2}|Z_{X_{2}}>z_{1-\alpha_{2}})$, will be truncated, where $Z_{X_{2}}$ denotes the standardized difference of the early‐phase Phase II trial outcome. If the analysis of Phase III data occurs independently from earlier Phase trial data, we expect no increase of Type I error and bias, though the power might remain low due to the limited trial sample size. In the retrospective assessment of the totality of evidence in this rare disease setting, however, positive results from both the Phase II trial and Phase III trial may well be seen as reinforcing. This informally combines evidence between trials which often results in positively biased inferences in favor of the late‐phase treatment effect *b*_*y*_, while an error inflation is observed in the double‐regression late‐phase outcome inference (models (m2) and (m3)) (Figure 2). In such situations, the bias on ${\hat{b}}_{y}$ estimate, based on model (m2) is given by the following approximation (Appendix B),

(eq2) $$\text{Bias}({\hat{b}}_{y})=\sigma_{y}^{\prime }\frac{w_{2}\rho \lambda \sigma_{x2}}{\sigma_{x}^{\prime }\sqrt{n_{2}/2}},$$

where $\sigma_{y}^{\prime 2}=\sigma_{y}^{2}+\gamma^{2}D$, $\sigma_{x}^{\prime 2}=\sigma_{x}^{2}+D$, $\lambda =\frac{\phi (\omega )}{1-\Phi (\omega )}$, $\omega =\frac{z_{1-\alpha_{2}}-\mu_{x2}}{\sigma_{x2}/\sqrt{n_{2}/2}}$, *w*_2_ = *n*_2_/*n*. ϕ and Φ are probability density and cumulative functions of the standard normal distribution, respectively, $D=w_{2}((2\sigma_{x2}^{2}/n_{2})\zeta +A^{2}(1-w_{2}^{2}-w_{3}^{2})+2A(\mu_{x2}-\mu_{x}))$, $A=(\sigma_{x2}/\sqrt{n_{2}/2})\lambda$, $\zeta =\alpha_{2}\lambda -{\left(\lambda \right)}^{2}$.

An approximate value for $\text{MSE}({\hat{b}}_{y})$ of the double‐regression is equal to (Appendix B)

(eq3) $$\text{MSE}({\hat{b}}_{y})=\underset{\text{Bias}{\left({\hat{b}}_{y}\right)}^{2}}{\underbrace{2\sigma_{y}^{\prime 2}{\left(\frac{w_{2}\rho \lambda \sigma_{x2}}{\sigma_{x}^{\prime }\sqrt{n_{2}}}\right)}^{2}}}+\underset{\mathrm{Var}({\hat{b}}_{y})}{\underbrace{2\sigma_{y}^{\prime 2}\left(\frac{1-\rho^{2}}{n_{3}}+\frac{\rho^{2}}{n_{}}\right)}}.$$

As we observe in (eq3), the inflation in MSE depends on (i) the decision threshold to initiate the Phase III trial through λ parameter, (ii) the Phase II early‐phase outcome mean ($\mu_{x2}$) and variance $(2\sigma_{x2}^{2}/n_{2}$), (iii) the number of patients in the Phase II trial (*n*_2_) and (iv) and the magnitude of the correlation (ρ). An increase in $\sigma_{x2}$ results in an increase of MSE, while as *n*_2_ decreases, the MSE increases as well. A similar behavior is observed in terms of Type I error (Figure 2). More specifically, Type I error rates increase considerably with higher ρ, while the power curves, in general, increase with more patients being allocated to the Phase III trial (*n*_3_) (Figure 2).

Based on the aforementioned bias and mean square error expressions and by replacing parameters with their estimates, the late‐phase outcome effect and variance of a (bias) corrected *double‐regression* (DRC) model are estimated as (Appendix B),

(m4) $$\begin{array}{ll} {\hat{b}}_{y}^{\prime } & ={\hat{b}}_{y}-\tilde{\text{Bias}}({\hat{b}}_{y})\\ \tilde{\mathrm{var}}{\left({\hat{b}}_{y}\right)}^{\prime } & =2(s_{y}^{2}-{\hat{\gamma }}^{2}\hat{D})(\frac{1-{\hat{\rho }}^{2}}{n_{2}}+\frac{{\hat{\rho }}^{2}}{n_{}}). \end{array}$$

The above expressions hold when treatment arms within studies are equal. Nonetheless, similar analytical expressions for unequal within study allocation ratios, can be acquired by appropriately changing the variances of ${\hat{b}}_{x3},{\hat{b}}_{x2}$ in Appendix B.1 based on the treatment arms sample sizes. For example, if the allocation ratio between arms in the Phase II trial equals to 1:2, then the Phase II early‐phase endpoint variance increases, $9\sigma_{x}^{2}/2N_{2}$ and the introduced bias could be reduced by half.

## 4 BIAS REDUCTION BY ACCOUNTING FOR BETWEEN‐TRIAL EARLY‐PHASE OUTCOME VARIABILITY

All models above, including the bias corrected model, assume that the true overall treatment effect remains common between trials, no between‐study variability on the early and late‐phase outcomes exist and therefore, all *N* observations are derived from the same population. Phase II vs Phase III trials typically do not have similar protocols, as the Phase II trials are usually more restrictive in patient inclusions, therefore, exploring between‐study variability becomes relevant.

The decision‐induced bias discussed in Section 3, would materialize as difference in treatment effects between the two available trials as well. Therefore, accounting for between‐study variability may act as a less rough approach to minimize this decision‐induced bias. A proper estimation of the between‐trial early‐phase outcome variance is not feasible with just two available studies,^14^, ^15^, ^16^, ^17^ therefore, in this article we choose not estimate but only account for this variance to aid towards the reduction of the bias.

To achieve this, we utilize a mechanism based on power priors to account for the between‐study differences within a Bayesian framework.^18^ By estimating a power parameter $\hat{\eta }$ that represents conflict between the early‐phase outcome data of the two available trials, model (m3) can be further extended to account for the early‐phase outcome effect excess between‐trial variability, along with any other biases.^18^, ^19^, ^20^

### 4.1 Bayesian flexible double‐regression

Let us assume that data *X*_2_ exist for the early‐phase outcome from the Phase II study and B are a set of linear regression parameters. Given the definition of a power prior,^21^ the posterior distribution after observing the Phase II early‐phase outcome data would be

$$\begin{array}{ll} \pi (B|X_{2},\eta )\propto L{\left(B|X_{2}\right)}^{\eta }\pi_{0}(B). & \end{array}$$

Then, the posterior for B after observing the Phase III study's early‐phase outcome data (*X*_3_) would be

$$\begin{array}{ll} \pi (B|X,\eta )\propto L(B|X_{3})L{\left(B|X_{2}\right)}^{\eta }\pi_{2}(B). & \end{array}$$

The posterior distribution of $B|X_{2}$ in the normal case^22^ is known to be equal to

(eq4) $$\begin{array}{ll} B|X_{2},\eta & ∼N({\left(T_{2}^{\prime }T_{2}\right)}^{-1}T_{2}^{\prime }Y_{2},\frac{\sigma_{x}^{2}}{\eta }{\left(T_{2}^{\prime }T_{2}\right)}^{-1}), \end{array}$$

where **T**_2_ is the design matrix with column vectors **1**, **t**, and dimensions *N*_2_ · 2. We now consider the following conditional model for the early and late‐phase outcome data of *N*_3_ patients

(m5) $$\begin{array}{ll} X_{3}|t & ∼N(a_{x}+b_{x}t,\sigma_{x}^{2})\\ a_{x} & ∼N(\mu_{ah},\sigma_{ah}^{2}/\hat{\eta }),b_{x}∼N(\mu_{bh},\sigma_{bh}^{2}/\hat{\eta })\\ \sigma_{x}^{2} & ∼\text{IG}(\alpha_{h},\beta_{h})\\ Y_{3}|t,x_{3} & ∼N(a_{y}+b_{y}t+\rho \frac{\sigma_{y}}{\sigma_{x}}x_{3},(1-\rho^{2})\sigma_{y}^{2})\\ a_{y} & ∼N(0,10^{2}),b_{y}∼N(0,10^{2})\\ \sigma_{y}^{2} & ∼\text{IG}(1,1)\\ \rho & ∼U(-1,1). \end{array}$$

The conditional set‐up of model (m5) remains similar to (m3). Now dynamic informative power priors parametrized through $\hat{\eta }$ are placed on the early‐phase endpoint's parameters *a*_*x*_ and *b*_*x*_. Such priors control the borrowing of the historical data and discount the early‐phase prior in case of treatment effect's disagreement. We chose to model the parameters univariately to aid any formulation of elicited informative priors on *a*_*y*_, *b*_*y*_, and ρ, though, a wishart prior on the covariance matrix ∑ (m1) could have jointly accounted for the association between the model parameters.

#### 4.1.1 Estimation of η

**FIGURE D1:**
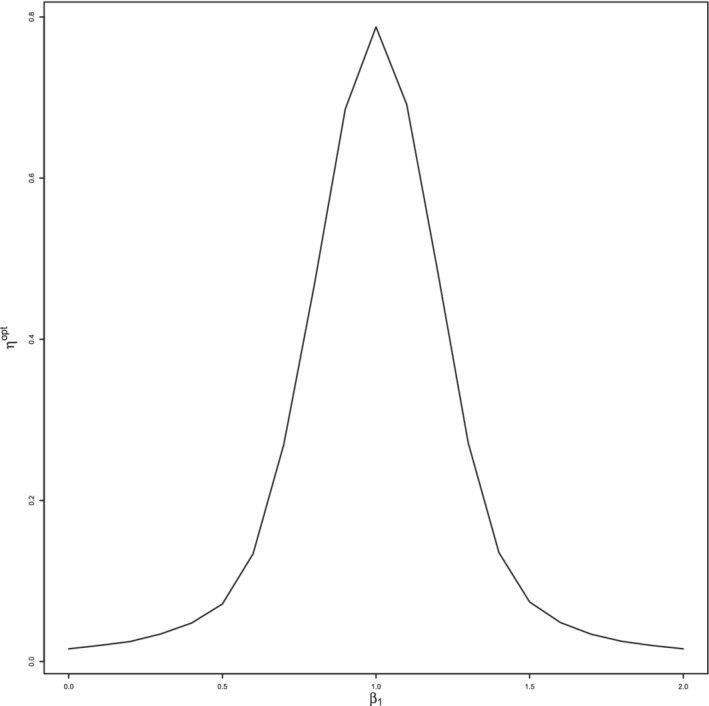
Relation between $\eta^{opt}$ and varying true *b*_*x*2_ when *b*_*x*3_ = 1 and $\sigma_{x}=\sigma_{y}=1$

A number of power prior (guided‐value) formulations has been suggested.^18^, ^19^, ^20^ Among the above alternatives, we chose one that selects a guided‐value that maximizes the marginal likelihood.^20^ The guide value of η based on the marginal likelihood criterion has an estimate of

(eq5) $$\hat{\eta }=\underset{0<\eta \leq 1}{\text{arg min}}[-2\log\{m(\eta )\}],$$

where m(η) is the marginal likelihood. Ibrahim et al^22^ provided an analytical expression of −2log{m(η)} for the normal outcome case. Figure D1 (Appendix D) presents the empirically calculated relationship between η and varying levels of *b*_*x*_.

In model (m5), similarly to model (m3), we are interested in the late‐phase overall primary outcome effect *b*_*y*_ and we assume that hypothesis testing for *H*_0_ vs *H*_1_ will be performed by utilizing posterior probabilities as $Pr(b_{y}>0|Y)>\omega$ where ω=0.95.

## 5 SIMULATION STUDY

The main four approaches discussed are summarized in Table 2. The corrected double‐regression approach as shown in Section 3 can be considered a rough (approximate) approach to minimize the decision‐induced bias. The Bayesian flexible double‐regression approach minimizes this bias by accounting for between‐trial differences without ad hoc corrections. Their relative performance in the analysis of the Phase III late‐phase outcome data, also in comparison to the two more trivial approaches (single and double‐regression) is the main focus of the simulation study.

**TABLE 2:** Summary of aforementioned statistical methods

Abbreviation	Model	(F)requentist/ (B)ayesian	Early/late‐phase	Phase (II/III)
(B)SR	(Bayesian) single‐regression	F/B	Late phase	III
(B)DR	(Bayesian) double‐regression	F/B	Early and late phase	II+III
DRC	Double‐regression corrected	F	Early and late phase	II+III
BFDR	Bayesian flexible double‐regression	B	Early and late phase	II+III

For illustrative purposes, we assume that the two available Phase II and Phase III trials had a similar control treatment, therefore, the Phase III trial would have been designed as a placebo‐controlled trial. In this section, we assume that the decision to conduct the Phase III trial was taken on the basis of available evidence in the first Phase II trial on a single early‐phase outcome. At the end of the Phase II trial, individual data of *N* patients are available on the early‐phase and data of *N*_3_ are available on the late‐phase outcomes. The simulation study results were derived from a bivariate normal model simulation strategy as described in Appendix C.

The SR, DR, DRC methods ignore any between‐study variability and therefore assume a different underlying data generating model in comparison to the Bayesian flexible *double‐regression* (BFDR) approach. Even though, they are not directly comparable (Table 2), we empirically compared the four aforementioned statistical methods by generating at least 10 000 simulated combinations of the two available trials data. To do so, we simulated scenarios of the final trial analysis on the late‐phase primary endpoint assuming a variety of combinations between the early‐phase (*b*_*x*_) and late‐phase (*b*_*y*_) outcome treatment effects. The latter were varied as (Scenario I) *b*_*y*_ = *b*_*x*_ = 0, (Scenario II) *b*_*y*_ = *b*_*x*_ = 0.6, (Scenario III) *b*_*x*2_, *b*_*y*2_ = 0,  *b*_*x*3_, *b*_*y*3_ = 0.2, and (Scenario IV) *b*_*y*_ = 0.6, *b*_*x*_ = 0, we assumed that ρ=0.9, $\alpha_{2}\in \{0.05,0.1,0.2\}$ the alpha level of the early‐phase primary outcome of Phase II trial, while all within‐study variances were set equal to 1. In the simulation setup we introduce a simulative parameter that place additional between‐trial variance on the early‐phase ($\tau_{x}$) and late‐phase ($\tau_{y}$) outcomes (see Appendix C for details). Specific alternative versions of scenarios I and II were produced by varying ρ and $\tau_{y},\tau_{x}$.

The first (I) scenario describes variations of the strict null ($\tau_{y}=\tau_{x}=0$) and null hypothesis with additional between‐trial variance ($\tau_{y}=\tau_{x}=0.3$), while the second (II) scenario describes a common alternative hypothesis on both outcomes and trials. Scenario III can occur when heterogeneous populations are selected for the Phase II and Phase III trial, while the fourth (IV) scenario describes a situation where the late‐phase outcome true effect exists but the early‐phase outcome equals to 0. All remaining settings (ie. number of trials (*k*), total sample sizes *N*, sample size ratio between trials *N*_2_ : *N*_3_, within‐study allocation ratios *n*_*ck*_ : *n*_*ek*_) were reflective of a typical rare disease setting and based on the Galafold example (Table 1). All simulations were performed via R^23^ and JAGS.^13^

### 5.1 (Strict) null hypothesis scenario (I: *b*_*y*_ = *b*_*x*_ = 0)

The BFDR results in treatment effects closer to the SR estimates than the DR approach under the null hypothesis simulation (Scenario I—Table 3). The DRC approach presents a similar behavior producing late‐phase effect estimates even closer to the SR than the BFDR approach. In the three null hypothesis scenarios I(b‐d) (*b*_*y*_ = *b*_*x*_ = 0), DR results in the largest estimated treatment effect and produces the largest type I error inflation while DRC generally inflates the Type I error the least among the three investigated methods. An interesting exception that we further discuss in Section 7, is observed in scenario Ia, where the BFDR approach produces stricter error rates than the DRC approach. In general, the SR method controls type I error the most, while the DR method controls type I error the least. The DR and DRC methods consistently produce the smallest C(r)Is, while the BFDR method produces the largest C(r)Is among the investigated methods.

**TABLE 3:** Late‐phase conditional average treatment effect estimates (means, posterior means, confidence intervals, credible intervals) and average treatment efficacy *P*‐values and probabilities of the four models (Table 2) given that ρ=0.9, $\tau_{x}=\tau_{y}=0.01$, and $\sigma_{y}=\sigma_{x}=1$, except where noted otherwise, based on at least 10.000 simulations

Scenario	Model		Mean/Posterior mean *b*_*y*_	Type I error	C(r)I widths
			$\alpha_{2}$: (0.05 · 0.1 · 0.2)	$\alpha_{2}$: (0.05 · 0.1 · 0.2)	$\alpha_{2}$: (0.05 · 0.1 · 0.2)
Ia. *b*_*y*_ = *b*_*x*_ = 0	SR		0.001 · 0.003 · 0.002	0.057 · 0.054 · 0.053	1.138 · 1.138 · 1.136
	DR		0.256 · 0.220 · 0.178	0.318 · 0.247 · 0.183	0.808 · 0.810 · 0.811
	DRC		0.087 · 0.075 · 0.063	0.079 · 0.066 · 0.060	0.810 · 0.812 · 0.813
	BFDR		0.170 · 0.156 · 0.133	0.054 · 0.037 · 0.022	1.343 · 1.330 · 1.319
b. *b*_*y*_ = *b*_*x*_ = 0	SR		0.000 · 0.003 · 0.002	0.055 · 0.053 · 0.054	1.138 · 1.138 · 1.138
ρ=0.5	DR		0.141 · 0.123 · 0.100	0.148 · 0.130 · 0.114	1.010 · 1.010 · 1.011
	DRC		−0.028 · −0.022 · −0.015	0.045 · 0.047 · 0.048	1.012 · 1.012 · 1.012
	BFDR		0.089 · 0.083 · 0.071	0.070 · 0.066 · 0.056	1.211 · 1.206 · 1.203
c. *b*_*y*_ = *b*_*x*_ = 0	SR		0.002 · 0.004 · 0.002	0.058 · 0.054 · 0.054	1.188 · 1.187 · 1.187
$\;\tau_{x}=\tau_{y}=0.3$	DR		0.246 · 0.211 · 0.171	0.267 · 0.211 · 0.164	0.896 · 0.898 · 0.899
	DRC		0.006 · 0.007 · 0.009	0.041 · 0.042 · 0.042	0.883 · 0.885 · 0.887
	BFDR		0.136 · 0.126 · 0.109	0.069 · 0.052 · 0.037	1.330 · 1.318 · 1.309
d. *b*_*y*_ = *b*_*x*_ = 0	SR		0.000 · 0.002 · 0.003	0.055 · 0.053 · 0.054	1.188 · 1.188 · 1.187
$\;\tau_{x}=\tau_{y}=0.3$	DR		0.135 · 0.117 · 0.097	0.139 · 0.122 · 0.110	1.067 · 1.068 · 1.068
ρ=0.5	DRC		0.002 · 0.004 · 0.006	0.059 · 0.058 · 0.059	1.064 · 1.065 · 1.065
	BFDR		0.073 · 0.069 · 0.060	0.076 · 0.072 · 0.065	1.209 · 1.205 · 1.201

*Note*: The first line SR of each scenario (I) presents a frequentist *single‐regression* on the Phase III late‐phase outcome data. DR correspond to the frequentist *double‐regression*. Last, the DRC lines present the result for the bias corrected *double‐regression* approach and the BFDR lines present the results for the Bayesian flexible *double‐regression* approach. $\alpha_{3}=0.05$ and $\alpha_{2}$ denotes the alpha level of the early‐phase primary outcome of the phase II trial.

### 5.2 Alternative hypothesis scenario (II: *b*_*y*_ = *b*_*x*_ = 0.6)

In scenario II (*b*_*y*_ = *b*_*x*_ = 0.6), all methods identified a treatment effect close to the true value (Table 4). The empirical power to identify a treatment effect is usually large for the BFDR, and considerably larger for the DRC than SR approach. Among the DRC and BFDR methods, BFDR produces treatment effect means closest to the true value. In scenario IIa ($\tau_{y}=\tau_{x}=0$), DRC performs better in terms of 95% coverage whereas in scenario IIb where $\tau_{y}=\tau_{x}=$ 0.3, BFDR results in coverage closest to 95%. The C(r)Is widths retained a similar behavior to the null hypothesis scenarios.

**TABLE 4:** Late‐phase conditional average treatment effect estimates (means, posterior means, confidence intervals, credible intervals) and average treatment efficacy *P*‐values and probabilities of the four models (Table 2) given that ρ=0.9, $\tau_{x}=\tau_{y}=0.01$, and $\sigma_{y}=\sigma_{x}=1$, except where noted otherwise, based on at least 10.000 simulations

Scenario	Model		Mean/Posterior mean *b*_*y*_	Power	95% coverage	C(r)I widths
			$\alpha_{2}$: (0.05 · 0.1 · 0.2)	$\alpha_{2}$: (0.05 · 0.1 · 0.2)	$\alpha_{2}$: (0.05 · 0.1 · 0.2)	$\alpha_{2}$: (0.05 · 0.1 · 0.2)
IIa. *b*_*y*_ = *b*_*x*_ = 0.6	SR		0.598 · 0.596 · 0.598	0.659 · 0.655 · 0.658	0.940 · 0.940 · 0.942	1.138 · 1.137 · 1.138
	DR		0.643 · 0.625 · 0.612	0.942 · 0.924 · 0.909	0.954 · 0.952 · 0.951	0.811 · 0.812 · 0.812
	DRC		0.634 · 0.621 · 0.611	0.935 · 0.920 · 0.907	0.956 · 0.954 · 0.952	0.812 · 0.812 · 0.813
	BFDR		0.632 · 0.617 · 0.607	0.663 · 0.634 · 0.612	0.997 · 0.997 · 0.997	1.304 · 1.304 · 1.305
b. *b*_*y*_ = *b*_*x*_ = 0.6	SR		0.598 · 0.596 · 0.598	0.626 · 0.624 · 0.625	0.940 · 0.941 · 0.942	1.188 · 1.187 · 1.188
$\;\tau_{x}=\tau_{y}=0.3$	DR		0.647 · 0.628 · 0.614	0.888 · 0.866 · 0.848	0.948 · 0.949 · 0.940	0.898 · 0.899 · 0.900
	DRC		0.634 · 0.621 · 0.612	0.876 · 0.859 · 0.845	0.950 · 0.949 · 0.946	0.896 · 0.898 · 0.899
	BFDR		0.629 · 0.615 · 0.607	0.648 · 0.622 · 0.610	0.989 · 0.989 · 0.990	1.292 · 1.293 · 1.293
III. *b*_*x*3_, *b*_*y*3_ = 0.2,	SR		0.202 · 0.204 · 0.202	0.173 · 0.169 · 0.168	0.941 · 0.941 · 0.945	1.188 · 1.187 · 1.187
*b*_*x*2_, *b*_*y*2_ = 0	DR		0.363 · 0.328 · 0.289	0.470 · 0.399 · 0.337	0.906 · 0.931 · 0.950	0.894 · 0.896 · 0.898
$\tau_{x}=\tau_{y}=0.3$	DRC		0.226 · 0.221 · 0.214	0.244 · 0.232 · 0.223	0.961 · 0.963 · 0.968	0.883 · 0.886 · 0.889
	BFDR		0.315 · 0.296 · 0.271	0.194 · 0.158 · 0.125	0.985 · 0.987 · 0.991	1.307 · 1.299 · 1.296
IV. *b*_*y*_ = 0.6, *b*_*x*_ = 0	SR		0.602 · 0.602 · 0.602	0.626 · 0.626 · 0.630	0.941 · 0.941 · 0.945	1.188 · 1.188 · 1.187
$\tau_{x}=\tau_{y}=0.3$	DR		0.846 · 0.846 · 0.771	0.988 · 0.988 · 0.971	0.828 · 0.828 · 0.906	0.896 · 0.896 · 0.899
	DRC		0.606 · 0.606 · 0.609	0.870 · 0.870 · 0.869	0.960 · 0.960 · 0.967	0.883 · 0.883 · 0.887
	BFDR		0.735 · 0.735 · 0.708	0.736 · 0.736 · 0.743	0.970 · 0.971 · 0.985	1.329 · 1.330 · 1.309

*Note*: The first line SR of each scenario (II,III,IV) presents a frequentist *single‐regression* on the Phase III late‐phase outcome data. DR correspond to the frequentist *double‐regression*. Last, the DRC lines present the result for the bias corrected *double‐regression* approach and the BFDR lines present the results for the Bayesian flexible *double‐regression* approach. $\alpha_{2}$ denotes the alpha level of the early‐phase primary outcome of the phase II trial. In Scenario III the correction for the DRC method is calculated based on that the true late‐phase outcome effect is equal to 0.2.

### 5.3 Scenarios III and IV

In scenario III (*b*_*y*2_ = 0, *b*_*y*3_ = 0.2, *b*_*x*2_ = 0, *b*_*x*3_ = 0.2), the BFDR produces similar findings to the DR approach, while the DRC method discards most Phase II information and its results are close to the SR approach (Table 4). DRC retains a comparable behavior in scenario IV (*b*_*y*_ = 0.6, *b*_*x*_ = 0), where it discards most of the decision‐induced bias and it produces results closer to the analysis of the Phase III study alone. In scenarios III, IV, as well as I, the naive pooling represented via the formal DR method, systematically and largely overstates our confidence in treatment efficacy.

### 5.4 Summary of simulation results

Among the four methods, the *single regression* performed best in terms of type I error followed closely by the DRC. Similarly, the approach that led to the least bias was the SR, again followed closely by the DRC. The DRC and DR methods resulted in the narrowest intervals. The intervals of the BFDR were comparable or larger than these of the SR. In terms of power, the DR method showed the highest gain, closely followed by the DRC. Finally, the SR and DRC both attained coverage close to nominal levels.

Overall, the DRC resulted in similar operational characteristics to the SR but it demonstrated a large gain in empirical power under the alternative hypothesis scenarios in comparison to the SR (Tables 3 and 4).

## 6 DISCUSSION

In a drug development procedure, it is not uncommon that positive Phase II results on early‐phase (biomarker) outcomes are not predictive of a Phase III success on late‐phase clinical outcomes. If Phase II and Phase III results are then assessed (perhaps informally) jointly to support efficacy, this retrospective (ad hoc)) assessment may be subject to decision‐induced bias and may increase uncertainty of the true primary late‐phase treatment effect. Such an informal combination of results may increase to a great extent (more than three times) the Type I error rate of null hypothesis, rendering the retrospectively combined late‐phase true treatment effect misleading. Especially in rare diseases, where the validation of early‐phase surrogate endpoints can become problematic, due to the small and often heterogeneous populations, the small sample sizes and the insufficient number of available trials, only late‐phase hard endpoints are usually appropriate to prove treatment efficacy.

In this article, in addition to identifying and investigating the above issue, we explored methods that can be utilized in order for early and late Phase trial data to be combined retrospectively (ie, right before drug marketing authorization request), while accounting for the underlying decision‐induced bias. The flexible BDR includes the borrowing of historical information, while this model downgrades the historical prior upon early‐phase outcome data conflict. The DRC method approximately corrects the biased late‐phase mean effect and variance estimate.

In most scenarios, the DRC method better controls the Type I error and bias than the DR and BFDR methods. This is not observed in scenario Ia, where the BFDR controls better the Type I error than the DRC. This possibly happens because the BFDR approach completely downgrades the impact of Phase II trial when its early‐phase treatment effect is different than the Phase III trial early‐phase treatment effect. Therefore, on average the Bayesian approach becomes less prone to false‐positive results based on possible very positive Phase II early‐phase outcome trial effects when $\tau_{x}$ is low and/or ρ is high (see, black dots of inner right panel of Figure 2). On the contrary, the DRC corrects the Phase II effect and then utilizes both Phase II and Phase III effects without heavily downgrading the Phase II results data upon data conflict. The DRC requires a known $\alpha_{2}$ but despite being approximate, it applies a more direct (decision‐based) penalty to the Phase II effect than the Bayesian approach; which could explain its overall better performance in the simulation.

Both the BFDR and the DRC methods would be an attractive solution to the increased Type I error of the informal retrospective combination of two small available trials. The consideration of these methods was shown to be rather important when, (i) the preceding Phase II trial conservatively (ie, alpha level was small) resulted to the Phase III trial and/or (ii) the association of utilized early and late‐phase outcomes is high. An informal combination of results across Phases often happens when both of the above hold, though, when neither holds then the complexity of suggested methods may outweigh the gains of their application.

Alternative versions of the BFDR model could be developed and they may perform more optimally in comparison to the current (ie, in terms of controlling the overall type I error) when applied on the flexible BDR via the use of an alternative guided value.^18^, ^19^, ^20^ The power parameter is imposed on the early‐phase endpoint and only indirectly affects the primary late‐phase endpoint, therefore, inference on the late‐phase endpoint via alternative guided values on the early‐phase endpoints could be expected to be more comparable to some extent.

An alternative approach that controls type I error on the late‐phase outcome, while borrowing historical information, may also provide a more formal solution.^19^ Future research could compare these alternatives vis‐á‐vis each other or with other methods. More covariates could be included, and then their performance could be tested with ease as all presented models are readily generalizable to full regressions. In this article, we set independent informative priors on the model parameters, however, accounting for the correlation between these parameters could also be considered through a well‐defined informative Wishart prior on the whole covariance matrix. Finally, in this work, we accounted for but did not estimate between‐study variance. Due to the only two available studies, a proper estimation of the between‐study outcome variability is currently known to be almost nonfeasible.^14^, ^15^, ^16^, ^17^

In the motivating example we assumed that both trials were superiority trials, while if we had kept the initial designs, different strategies may have been more appropriate. Nonetheless, examples of two superiority trials, one Phase II and one Phase III, exist in the literature. For example, the drug development program of thalidomide for the treatment of multiple myeloma contained two randomized superiority clinical studies of similar design, a supportive (GISMM2001) and a main study (IFM 99‐06), that compared melphalan‐prednisone (control treatment) to thalidomide (experimental treatment).^2^ The supportive study was shorter and it reported clinical response rates and event free survival as primary endpoints. The main study was longer in duration and it reported overall survival, as main endpoint and clinical response rates and event free survival, as secondary endpoints. The suggested methodology could be tailored to account for the possibility of decision‐induced bias under survival and other types of outcomes and even to combine different study designs.

Throughout the article normality was assumed, an assumption that could be challenged with rare diseases sample sizes.^1^, ^2^, ^3^ We approximated a truncated normal with a normal distribution with mean and variance equal to that of the former. This decision was made to aid calculations on the distribution mixture (Appendix B). Better approximations for the truncated normal distribution may exist, such as the chi‐square distribution and their performance could be explored as well.^24^ We should note that for moderately sized *N*_2_ in comparison to *N* and small correlation between the two outcomes, a SR might be more efficient than a DR, due to the noise introduced by the early‐phase outcome.^5^ In the simulation study we assumed that the Phase II trial had equal allocation between trial arms, while the Phase III trial had allocation equal to 1:2 between the control vs treatment arm. We expect that our findings would be comparable under different allocations between arm sample sizes, though further investigation could provide more insights between the relative performance of BDR and DRC methods.

In this article, we performed a post hoc (retrospective) combination of available information after the conduct of the Phase II and Phase III trial. However, it may be very relevant to (prospectively) plan to pool the data from both studies and to use the early‐phase outcomes of the Phase II study to increase the precision, with which the efficacy on late‐phase outcome is estimated overall.^7^, ^8^, ^9^ An alternative strategy could be to conduct one single trial with interim analysis, then, based on the observed treatment effects on the early‐phase endpoints decide whether to follow‐up the patients.^8^

To conclude, especially in a small population context, the often informal retrospective pooling of a single Phase II early‐phase outcome data to support the true late‐phase outcome data inference at the end of a single confirmatory Phase III trials could induce bias and it should be performed via formal numerical approaches. Such approaches should control this decision‐induced bias, in order to avoid inflating the Type I error under the null hypothesis and prevent overestimating our beliefs on the primary treatment effect. We hope that this article, except for introducing possible solutions, raises awareness of potential mishaps with post hoc combinations of trial outcome results.

## Figures and Tables

**FIGURE D2:**
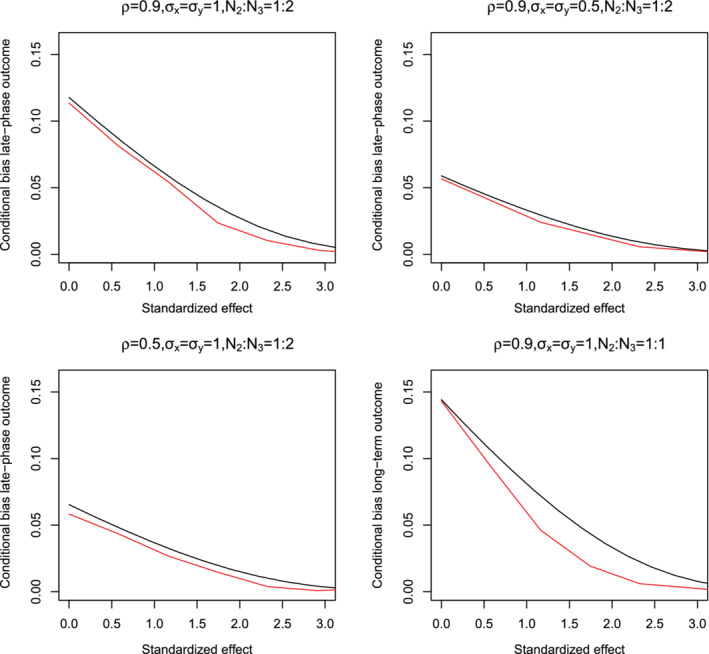
Approximation of a truncated normal distribution with a normal distribution ($\alpha_{2}=0.1$). The black lines represent the approximate analytical solution to the bias, while the red lines represent the simulated values based on the *double‐regression* model [Colour figure can be viewed at wileyonlinelibrary.com]

## Data Availability

The data that support the findings of this study are openly available in GitHub at https://github.com/kpatera/data‐earlylate.

## APPENDIX A DETAILS OF (BAYESIAN) UNIVARIATE MODEL

### A.1

The standard linear SR reference model to demonstrate late‐phase treatment efficacy assumes $Y|t∼N(a_{y}+b_{y}t,\sigma_{y}^{2})$, where $\sigma_{y}$ denotes the true outcome variance, **t** denotes a vector of length *n*_3_ indicating whether a patient receives control or experimental treatment.

A conjugate Bayesian analogue (BSR) of the model above can be expressed also as above where *a*_*y*_, *b*_*y*_, and $\sigma_{y}$ are random variables and need a prior distribution. This model offers the flexibility to directly impact inference via placing informative priors on parameters *a*_*y*_, *b*_*y*_, and $\sigma_{y}$. Model B(SR) corresponds exactly to the aforementioned model SR under convenient noninformative priors on *a*_*y*_, *b*_*y*_, and $\sigma_{y}$.^12^

In the above SR model, we are interested in ${\hat{b}}_{y}$ and we assume that hypothesis testing for *H*_0_ : *b*_*y*_ = 0 vs *H*_1_ : *b*_*y*_ > 0 will be evaluated as $z_{1-\alpha_{3}}<{\hat{b}}_{y}/\sqrt{\mathrm{var}({\hat{b}}_{y})}=\Phi ({\hat{b}}_{y}/\sqrt{\mathrm{var}({\hat{b}}_{y})})$, where $z_{1-\alpha_{3}}$ is the $\alpha_{3}th$ standard normal quantile. In the Bayesian SR analogue, we are interested in *b*_*y*_ and we assume that hypothesis testing for *H*_0_ vs *H*_1_ will be performed by utilizing posterior probabilities as $Pr(b_{y}>0|Y)>\omega$ where ω=0.95.

## APPENDIX B DERIVATION OF $\text{MSE}({\hat{b}}_{y})$

### B.1

The $\text{MSE}({\hat{b}}_{y})$ of the late‐phase outcome equals to

$$\begin{array}{ll} \text{MSE}({\hat{b}}_{y}) & =\text{Bias}{\left({\hat{b}}_{y}\right)}^{2}+\mathrm{Var}({\hat{b}}_{y}). \end{array}$$

#### B.1 Derivation of *Bias*(*b*_*y*_)

Let assume that $\sigma_{x2},\sigma_{x3}$ are known for the Phase II and Phase III trials, then the early‐phase outcome treatment effect estimates are distributed as ${\hat{b}}_{x3}∼N(\mu_{x3},\frac{2\sigma_{x3}^{2}}{n_{3}})$ and ${\hat{b}}_{x2}∼N(\mu_{x2},\frac{2\sigma_{x2}^{2}}{n_{2}})$. In practice the Phase II early‐phase outcomes would follow an one‐sided truncated normal distribution. The adjusted mean ($\mu_{x2}$) and variance ($\sigma_{x2}^{2}$) of this early‐phase outcome one‐sided truncated normal distribution ${\hat{b}}_{x2}∼N_{\alpha_{2}}(\mu_{x2}^{\prime },\frac{2\sigma_{x2}^{\prime 2}}{n_{2}})$ equal to

(eq3) $$\begin{array}{ll} \mu_{x2}^{\prime } & =\mu_{x2}+\frac{\sigma_{x2}}{\sqrt{n_{2}/2}}\lambda \end{array}$$

(eq4) $$\begin{array}{ll} \sigma_{x2}^{\prime 2} & =\sigma_{x2}^{2}[1+\zeta ], \end{array}$$

where $\lambda =\frac{\phi (\omega )}{1-\Phi (\omega )}$, $\zeta =a\lambda -(\lambda ){}^{2}$ and $\omega =Z_{1-\alpha_{2}}^{x}-\frac{\mu_{x2}^{\prime }}{\sigma_{x2}^{\prime }/\sqrt{n_{2}/2}}$ and ϕ and Φ are the probability density and the cumulative function of the standard normal distribution.

We assume that we can approximate a truncated normal with a normal distribution with updated mean and variance as follows ${\hat{b}}_{x2}\;\overset{\text{approx}}{∼}\;N(\mu_{x2}^{\prime },\frac{2\sigma_{x2}^{\prime 2}}{n_{2}})$.^25^ The overall ${\hat{b}}_{x}$ would be a mixture of the above density functions.

Given the set of two densities and weights (*w*_1_ and *w*_2_), such that *w*_*i*_ ≤ 0 and $\sum w_{i}=1$ the mixture can be represented as

$$\begin{array}{ll} f(x)=\overset{2}{\underset{k=1}{\sum }}w_{k}p_{k}(x). & \end{array}$$

The mean and variance of the above normal mixture of two distributions equal to $\mu_{x}=\sum_{k=1}^{2}w_{k}\mu_{xk}$ and $\sigma_{x}^{2}=\sum_{k=1}^{2}w_{k}(\mu_{xk}^{2}+\frac{2\sigma_{xk}^{2}}{n_{i}}-\mu_{x}^{2})$ with *w*_*k*_ = *N*_*k*_/*N*.^26^ Therefore, ${\hat{b}}_{x}∼N(\mu_{x},\sigma_{x}^{2})$.

Therefore, the updated mean and variance of ${\hat{b}}_{x}$, are equal to

(m6) $$\begin{array}{ll} \mu_{x}^{\prime } & =w_{2}\mu_{x2}^{\prime }+w_{3}\mu_{x3}\\ & =w_{2}\mu_{x2}+w_{3}\mu_{x3}+w_{2}\lambda \;\frac{\sigma_{x2}}{\sqrt{n_{2}/2}}\\ & =\mu_{x}+w_{2}\lambda \;\frac{\sigma_{x2}}{\sqrt{n_{2}/2}}\\ \sigma_{x}^{\prime 2} & =\overset{2}{\underset{k=1}{\sum }}w_{k}(\mu_{xk}^{2}+\frac{2\sigma_{xk}^{2}}{n_{k}}-\mu_{x}^{2})+D\\ & =\sigma_{x}^{2}+D, \end{array}$$

where $D=w_{1}((2\sigma_{x2}^{2}/n_{2})\zeta +A^{2}(1-w_{2}^{2}-w_{3}^{2})+2A(\mu_{x2}-\mu_{x}))$, $A=(\sigma_{x2}/\sqrt{n_{2}/2})\lambda$ and $\zeta =a\lambda -(\lambda ){}^{2}$.

A bias is introduced after combining the Phase II and III trial early‐phase outcome effect estimates as $\frac{\sigma_{x2}\lambda \cdot w_{2}}{\sqrt{n_{2}/2}}$.^26^ Then based on (eq1) and assuming that $\sigma_{x}=\sigma_{y}=1$, the bias of *b*_*y*_ equals to

(m7) $$\begin{array}{ll} Bias(B)=\frac{w_{2}\lambda \;\rho \sigma_{x2}}{\sqrt{n_{2}/2}}. & \end{array}$$

#### B.2 Derivation of $\mathrm{Var}({\hat{b}}_{y})$

The variance of late‐phase outcome *b*_*y*_ is equal to Reference,^5^

(eqA1) $$\mathrm{Var}(b_{y})=\mathrm{var}({\hat{b}}_{0})+{\hat{\gamma }}^{2}\mathrm{var}({\hat{b}}_{x})+{\hat{b}}_{x}^{2}\mathrm{var}(\hat{\gamma })+2{\hat{b}}_{x}\text{cov}({\hat{b}}_{0},\hat{\gamma }).$$

An estimate of $\mathrm{Var}({\hat{b}}_{y})$ can be obtain via estimates of the relevant parameters which can be obtained directly via the regression of *X*|**t** and the regression of *Y*|*X*, **t** on *N* and *N*_3_ patients, respectively.

Assuming that **t** is an indicator variable and *n*_*k*_ corresponds to the total sample size per treatment arm of the *kth* trial, the q‐dependent variance of (${â}_{x},{\hat{b}}_{x}$) can be derived as $\sigma_{x}^{\prime 2}{\left(T^{\prime }T\right)}^{-1}$, where *T* is the design matrix of *X*|**t** on *N* patients as follows

(eqA2) $$\begin{array}{ll} {\left(T^{\prime }T\right)}^{-1} & ={\left(\begin{array}{cc} 2n & n\\ n & n \end{array}\right)}^{-1}=\left(\begin{array}{cc} \left(1/n\right) & -(1/n)\\ -(1/n) & \left(2/n\right) \end{array}\right)=\frac{1}{n}\left(\begin{array}{cc} 1 & -1\\ -1 & 2 \end{array}\right) \end{array}$$

and as a mixture of two distributions $\sigma_{x}^{\prime 2}=\sigma_{x}^{2}+D$

From (eqA2), $\mathrm{var}(b_{x})=\frac{2\sigma_{x}^{\prime 2}}{n_{}}$, an estimate of which can be derived as $\hat{\mathrm{var}}({\hat{b}}_{x})=\frac{2s_{x}^{\prime 2}}{n_{}}$, where $s_{x}^{2}$ follows from the regression of *X*|**t** on *N* patients.

Subsequently, the variance of (${â}_{0},{\hat{b}}_{0},\hat{\gamma }$) can be derived as $\sigma_{o}^{2}(1-\rho^{2}){\left({T_{3}}^{\prime }T_{3}\right)}^{-1}$, where **T**_**3**_ is the design matrix of *Y*|*X*, **t** on *N*_3_ patients.

(eqA3) $$\begin{array}{ll} E(T_{3}^{\prime }T_{3})=\; & E\left(\begin{array}{ccc} 2n_{3} & n_{3} & \underset{C,E}{\sum }x_{3}\\ n_{3} & n_{3} & \underset{E}{\sum }x_{3}\\ \underset{C,E}{\sum }x_{3} & \underset{E}{\sum }x_{3} & \underset{C,T}{\sum }x_{3}^{2} \end{array}\right)=N_{3}\left(\begin{array}{ccc} 2 & 1 & \mu_{C}+\mu_{E}\\ 1 & 1 & \mu_{E}\\ \mu_{C}+\mu_{E} & \mu_{E} & 2\sigma_{y_{3}}^{2}+(\mu_{C}^{2}+\mu_{E}^{2}) \end{array}\right). \end{array}$$

The variance estimates are derived by inverting matrix (eqA3) and replacing $\sigma_{o}^{2}$ with $\sigma_{y}^{\prime 2}=\sigma_{0}^{2}+\gamma^{2}\sigma_{x}^{\prime 2}$.^8^

(eqA4) $$\mathrm{var}(\hat{\gamma })=\frac{\sigma_{y}^{\prime 2}(1-\rho^{2})}{2n_{3}\sigma_{x}^{\prime 2}},$$

(eqA5) $$\mathrm{var}({\hat{b}}_{0})=\frac{2\sigma_{y}^{\prime 2}(1-\rho^{2})}{n_{3}}+\frac{\sigma_{y}^{\prime 2}(1-\rho^{2})}{2n_{3}\sigma_{x}^{\prime 2}}b_{x}^{2},$$

(eqA6) $$\text{cov}({\hat{b}}_{0},\hat{\gamma })=-\frac{\sigma_{y}^{\prime 2}(1-\rho^{2})}{2n_{3}\sigma_{x}^{\prime 2}}b_{x}.$$

Replacing (eqA4) to (eqA6) in (eqA1) we obtain $\mathrm{var}({\hat{b}}_{y})$

$$\begin{array}{llll} \text{Var}({\hat{b}}_{y})=\; & \mathrm{var}({\hat{b}}_{0})+{\hat{\gamma }}^{2}\mathrm{var}({\hat{b}}_{x})+{\hat{b}}_{x}^{2}\mathrm{var}(\hat{\gamma })+2{\hat{b}}_{x}\text{cov}({\hat{b}}_{0},\hat{\gamma })\\ & \times \frac{2\sigma_{y}^{\prime 2}(1-\rho^{2})}{n_{3}}+\frac{\sigma_{y}^{\prime 2}(1-\rho^{2})}{2n_{3}\sigma_{x}^{\prime 2}}b_{x}^{2}+\frac{2\sigma_{y}^{\prime 2}\rho^{2}}{n_{}} & & \\ & +b_{x}^{2}\frac{\sigma_{y}^{\prime 2}(1-\rho^{2})}{2n_{3}\sigma_{x}^{\prime 2}}+2{\hat{b}}_{x}(-\frac{\sigma_{y}^{\prime 2}(1-\rho^{2})}{2n_{3}\sigma_{x}^{\prime 2}}b_{x})\\ = & \;2\sigma_{y}^{\prime 2}(\frac{\left(1-\rho^{2}\right)}{n_{3}}+\frac{\rho^{2}}{n_{}})\\ \sigma_{y}^{\prime 2}= & \;\sigma_{y}^{2}+\gamma^{2}\;D. \end{array}$$

#### B.3 Derivation of $\text{MSE}({\hat{b}}_{y})$

Based on the calculated alternative variance of the overall late‐phase effect $\mathrm{Var}({\hat{b}}_{y})$ and the method of moments, the $\text{MSE}({\hat{b}}_{y})$ is given by

$$\begin{array}{ll} \text{MSE}({\hat{b}}_{y}) & =Bias{\left({\hat{b}}_{y}\right)}^{2}+\mathrm{Var}({\hat{b}}_{y})\\ & ={\left(\frac{w_{2}\lambda \;\rho \sigma_{y}^{\prime }\sigma_{x2}}{\sigma_{x}^{\prime }\sqrt{n_{2}/2}}\right)}^{2}+2\sigma_{y}^{\prime 2}(\frac{1-\rho^{2}}{n_{3}}+\frac{\rho^{2}}{n_{}})\\ & =2\sigma_{y}^{\prime 2}{\left(\frac{w_{2}\rho \lambda \sigma_{x2}}{\sigma_{x}^{\prime }\sqrt{n_{2}}}\right)}^{2}+2\sigma_{y}^{\prime 2}(\frac{1-\rho^{2}}{n_{3}}+\frac{\rho^{2}}{n_{}})\\ \sigma_{y}^{\prime 2} & =\sigma_{y}^{2}+\gamma^{2}D. \end{array}$$

In Appendix D, Figure D2 presents a short simulation demonstrating the association between the approximate analytical bias and the bias introduced by the use of the DR method. Equivalent simulations were performed for the updated variance parameters, all scenarios resulted in less than 10% difference between the approximate and analytical derived variances.

## APPENDIX C BIVARIATE NORMAL SIMULATION

### C.1

Regarding the bivariate normal simulation strategy, we generated a series of parallel‐group design randomized trials with two treatment groups (control (C) and treatment (E)). We assume that the outcome values for *ith* control individual and *kth* trial, for the early‐phase *m*_*Cxik*_ and late‐phase *m*_*Cyik*_ outcome are generated by a bivariate normal distribution as follows

$$\begin{array}{ll} \left(\begin{array}{c} m_{Cxik}\\ m_{Cyik} \end{array}\right) & ∼\text{BVN}\left[\begin{array}{cc} \left(\begin{array}{c} \mu_{Cxk}\\ \mu_{Cyk} \end{array}\right), & \sum_{C}=\left(\begin{array}{cc} \sigma_{Cxk}^{2} & \rho_{C}\sigma_{Cxk}\sigma_{Cxk}\\ \rho_{C}\sigma_{Cyk}\sigma_{Czk} & \sigma_{Cyk}^{2} \end{array}\right) \end{array}\right], \end{array}$$

where $\mu_{Cik}$ are the true treatment means for each endpoint in the control arm and $\sum_{C}$ is their covariance matrix, $\sigma_{Ck}^{2}$ are the variances of the early and late‐phase endpoints and $\rho_{C}$ is the correlation between these endpoints.

In a similar fashion we generate data outcome values for the *ith* treatment individual in the *kth* trial as follows

$$\begin{array}{ll} \left(\begin{array}{c} m_{Exik}\\ m_{Eyik} \end{array}\right) & ∼\text{BVN}\left[\begin{array}{cc} \left(\begin{array}{c} \mu_{Ezk}=\mu_{Cxk}+\theta_{x}\\ \mu_{Eyk}=\mu_{Cyk}+\theta_{y} \end{array}\right), & \sum_{T}=\left(\begin{array}{cc} \sigma_{Exk}^{2} & \rho \sigma_{Exk}\sigma_{Eyk}\\ \rho \sigma_{Exk}\sigma_{Eyk} & \sigma_{Eyk}^{2} \end{array}\right) \end{array}\right]. \end{array}$$

In order to incorporate between‐study variability $\tau^{2}$, we can further assume that $m_{i}∼N(\Delta ,\tau^{2})$.^27^

$$\begin{array}{ll} \left(\begin{array}{c} m_{Exik}\\ m_{Eyik} \end{array}\right) & ∼\text{BVN}\left[\begin{array}{cc} \left(\begin{array}{c} \mu_{Exk}=\mu_{Cxk}+\Delta_{x}\\ \mu_{Eyk}=\mu_{Cyk}+\Delta_{y} \end{array}\right), & \sum_{T}=\left(\begin{array}{cc} \sigma_{Exk}^{2}+\tau_{x}^{2} & \rho \sigma_{Exk}\sigma_{Eyk}+\tau_{x}\tau_{y}\rho_{B}\\ \rho \sigma_{Exk}\sigma_{Eyk}+\tau_{x}\tau_{y}\rho_{B} & \sigma_{Eyk}^{2}+\tau_{y}^{2} \end{array}\right) \end{array}\right]. \end{array}$$

$\rho_{B}$ parameter indicates how the early‐phase and late‐phase outcome are related across all available studies. In our framework we only have available summary value on both early and late‐phase outcomes from only a single Phase III trial. Therefore, for simplicity in the simulation study we assume that the between‐study correlation equals to zero $\rho_{B}=0$.

We applied an alternative model that generates data in two stages to check for results' robustness with no observed noticeable variations in relative performances.
